# A randomized controlled clinical trial on the impact of CCR5 blockade with maraviroc in early infection on T-cell dynamics

**DOI:** 10.1097/MD.0000000000005315

**Published:** 2016-11-04

**Authors:** Maile Y. Karris, Anya Umlauf, Florin Vaida, Douglas Richman, Susan Little, Davey Smith

**Affiliations:** a University of California San Diego; b Veterans Affairs Medical Center, San Diego, CA.

**Keywords:** acute infection, maraviroc, treatment intensification

## Abstract

**Background:**

Initiation of antiretroviral therapy (ART) in early HIV infection demonstrates clinical benefits including enhanced CD4^+^ T-lymphocyte recovery and minimization of the latent HIV reservoir. Whether ART intensification with CCR5 blockade provides additional benefits is unknown.

**Trial design:**

: This randomized controlled trial evaluated the impact of maraviroc (MVC) intensification in persons starting ART in acute and early HIV (AEH, within 3 months of estimated date of infection).

**Methods:**

Twenty persons in AEH in San Diego underwent double-blind randomization to receive either standard of care (SOC) ART or SOC + MVC to evaluate the hypothesis that early CCR5 blockage with a CCR5-containing ART regimen may provide immunologic and clinical benefit. The primary outcome of this study was the difference from baseline to week 48 in the proportion of CCR5^+^ CD4^+^ memory T cells. Blood was drawn at baseline and weeks 12, 24, and 48 to evaluate CCR5^+^ CD4^+^ and CD8^+^ T-cell dynamics using multicolor flow cytometry.

**Results:**

MVC intensification (n = 10) did not significantly alter CCR5^+^ T-cell dynamics at week 48 of study compared to SOC (n = 9) in this fully recruited study (mean 1.12 vs 0.63, t = 0.36, df = 16, *P* = 0.727). Exploratory analyses of additional T-cell subsets suggest that MVC intensification in AEH trended to early greater increases in naïve and activated and proliferating CD4^+^ T cells (*P* = 0.11, 0.19), and greater decreases in senescent memory CD4^+^ T cells (*P* = 0.06), but these differences did not remain by week 48. CD8^+^ T-cell evaluations did demonstrate trends to differences at week 48 with slower increases in naïve cells and slower decreases in activated memory cells (*P* = 0.16, 0.09). There were no reported harms or significantly different adverse events.

**Conclusions:**

We did observe a few trends, but did not find compelling evidence that MVC intensification during AEH significantly impacts CD4^+^ and CD8^+^ T-cell dynamics. Diagnosing and starting persons in AEH on ART may be of greater clinical importance than the regimen initiated.

## Abstract 2

Supplemental Digital Content is available in the text

## 1 Introduction

Starting antiretroviral therapy (ART) as early as possible into HIV infection has considerable clinical benefits. In fact, when ART is started in recent HIV infection, CD4 T cells are recovered to normal levels and HIV-associated inflammation is minimized.^[1]^

Shortly following infection with HIV, the virus rapidly replicates in gut-associated lymphoid tissue (GALT), and within a few weeks, the CD4^+^ T cell population in these tissues is depleted.^[2,3]^ Interestingly, the CD4^+^ T cell population of non-GALT lymphoid tissues remains largely intact.^[4]^ This cellular depletion in the gut during recent infection specifically occurs to CD4^+^ lymphocytes, which selectively express CCR5^+^ (usually activated memory and effector cells).^[5–8]^ These events may not be a coincidence given that most new infections are with HIV populations that use the CCR5 coreceptor. These observations have led to the theory that in regards to HIV pathogenesis, “the die is cast” in primary HIV infection,^[9]^ and perhaps the benefits observed in studies initiating ART during primary infection are the mitigation of these early events.^[1,10–12]^ The vast majority of patients with primary HIV-1 infection are infected with CCR5 tropic virus.^[13]^ We hypothesized that early CCR5 blockage with a CCR5-containing ART regimen may provide immunologic and clinical benefit. The objective of this study was to evaluate the impact of maraviroc (MVC), an antiretroviral medication that inhibits HIV cellular entry through the CCR5 coreceptor, intensification to a standard of care (SOC) antiretroviral regimen on proportions of CD4^+^ and CD8^+^ T-cell subsets in persons starting ART in early (<3 months from estimated date of infection [EDI]) HIV infection.

## 2 Methods

### 2.1 Population and design

This study was a double-blind, placebo-controlled, 1:1 randomized clinical trial comparing SOC ART (atazanavir, ritonavir, tenofovir, and emtricitabine) with SOC ART plus MVC. The protocol was reviewed and approved by the local ethics committee. The protocol for this study (ViiV-a-la-GALT) was reviewed and approved by the University of California San Diego Human Research Protections Program. The full trial protocol can be accessed by contacting the corresponding author.

We recruited 20 acute or very early HIV infected persons aged ≥18 years from August 2010 to March 2014 (when we reached our target enrollment number) in San Diego, CA, at the University of California San Diego's AntiViral Research Center. The primary eligibility criteria included persons in acute or early HIV infection using nucleic acid and detuned HIV-1 testing as previously described^[1]^ who did not demonstrate genotypic resistance to the study drug regimens. Prior to study launch, the study statistician generated the randomization schedule and this was provided to the study pharmacist who provided all study drugs. Each participant received their individual medication based on their randomization scheduled. This procedure is the standard used in the AIDS Clinical Trials Group. This procedure allows blinding of study participants, coordinators, investigators, and those performing data entry. Following randomization, study participants initiated SOC ART + once-daily placebo or SOC ART + once-daily MVC within 16 days of screening and were followed longitudinally at weeks 2, 4, 8, 12, 16, 24, 32, 40, and 48 and as needed. There were no changes to methods after trial commencement.

### 2.2 Study procedures

At study screening and before the initiation of ART, participants underwent a study entry evaluation (including past medical history), physical and laboratory assessments (including HIV viral loads [COBAS, Roche, Indianapolis, IN]), CD4^+^ counts, HIV-1 drug resistance testing by *pol* genotype (Genosense, Monogram Biosciences, San Francisco, CA), and coreceptor usage (Trofile, Monogram Biosciences). Whole blood was then drawn from study participants at weeks 0, 12, 24, and 48 and peripheral blood mononuclear cells (PBMCs) were isolated from plasma using gradient centrifugation. Isolated PBMCs were washed and aliquoted into tubes of 1 million PBMCs/200 μL staining buffer prior to incubation with conjugated antibodies (Becton, Dickinson and Company, Franklin Lakes, NJ): CD3 (APC-Cy7), CD4 (PerCP), CD8 (Pac-Blue), CD45RO (PE), CD27 (APC), CCR5 (FITC), CCR7 (PE-Cy7), CD38 (PE-Cy7), and HLA-DR (FITC). Conjugated antibodies to intracellular Ki67 (FITC) were used with fixation and permeability buffers (eBioscience, San Diego, CA) for intracellular staining assays per manufacturer instructions. Lymphocyte subsets were measured on a BD FACSCanto II instrument (BD Biosciences, San Jose, CA) and data analyzed with FlowJo software. One investigator implemented the gating strategy to ensure a consistent gating approach. For our primary outcome, CCR5 expression gating was set at the border of CD45RO^−^CD27^+^ cells that were not expected to express much (if any CCR5). An example of flow cytometry gating is provided in Supplemental Figure 1. Percentages were analyzed based on CD4 and CD8 T-cell lineages. Viral loads (Roche) were measured in collected blood plasma.

A proportion of study participants also underwent quantification of HIV DNA using digital droplet polymerase chain reaction as previously described.^[14]^

### 2.3 Study outcomes

The primary outcome of this study was the difference from baseline to week 48 in the proportion of CCR5^+^ CD4^+^ memory T cells. Exploratory outcomes included the difference in additional CD4^+^ and CD8^+^ T-cell subsets from baseline to week 12, baseline to week 24, and baseline to week 48.

### 2.4 Sample size

Based on the primary outcome of this study, with 10 patients per arm, the study was expected to have 80% power to detect a difference of 0.55 in the proportion of CCR5^+^ CD4^+^ memory T cells at 48 weeks after start of therapy between the 2 randomized treatment arms. This power calculation is based on the estimate of the standard deviation of the proportion of CCR5^+^ CD4^+^ memory cells at 48 weeks after start of therapy of 0.08.

### 2.5 Statistical analyses

All analyses were by original assigned groups. All statistical analyses were 2-sided and performed with a significance level of 0.05. Baseline demographic and medical characteristics were compared between arms using Wilcoxon rank-sum test and Fisher exact test for numeric and categorical predictors, respectively. Changes in primary and log-odds transformed secondary outcomes were calculated as differences in outcome values from baseline to weeks 12, 24, and 48 separately, where negative difference indicates decrease in values. Changes in primary outcome (CCR5^+^CD45RO^+^CD27^−^CD4^+^) were compared with the 2-sample *t* test (assumptions for parametric analyses were checked). Because group differences were found in levels of baseline CD4^+^ T cells, the primary analyses were followed by analysis of covariance to compare the primary outcome between arms while controlling for this covariate. Wilcoxon rank-sum procedure was used in analysis of the secondary outcomes. Although differences in log-odds of the secondary outcomes were used for the analyses per study protocol and the reported *P* values are from those analyses, the section Results reports median differences in untransformed values rather than log-odds transformations for ease of interpretation. Multivariate analyses were not pursued given the lack of significant results in univariate analyses.

## 3 Results

### 3.1 Description of study participants

**Figure 1:**
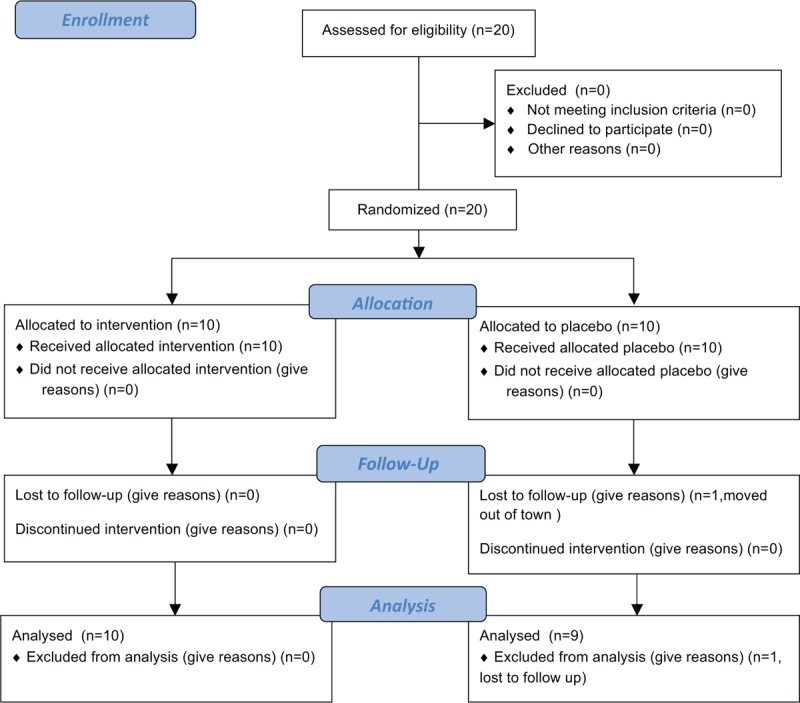
Study participant flow diagram demonstrating the numbers of participants randomly assigned and ultimately analyzed for the primary outcome.

Of the 20 persons enrolled (10 enrolled to intervention and 10 to placebo), 1 was lost to follow-up (moved from San Diego) and was not included in analyses (Fig. 1). All participants were men with a median age 30.5 years (28, 40 years). Participants were white (63%), Hispanic (21%), and Asian (16%). At baseline, they had a median CD4^+^ T-cell count of 559 cells/mL (428, 672 cells/mL), CD4 percentage of 31% (26, 33.5%), and HIV viral load of 5.07 log_10_ copies/mL (3.21, 5.99 log_10_ copies/mL). Participants were recruited with a median of 24 days (19, 76 days) from EDI and all initiated ART within 91 days (range 20–91 days) from EDI. There were no significant differences in most baseline characteristics including age, race, viral load, EDI, and time from infection to initiation of ART. Persons randomized to the MVC arm, however, did have significantly higher median CD4^+^ T cells, 708 cells/mL (511,815 cells/mL), compared to those in the SOC arm, 501 cells/mL (428,524 cells/mL), *P* = 0.045 (Table 1).

**Table 1:**
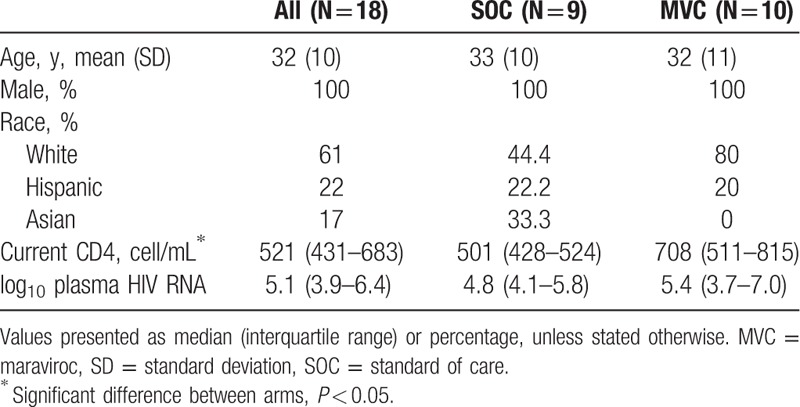
Baseline characteristics of the cohort.

There were no reported harms or unintended effects observed in this study.

### 3.2 The impact of MVC intensification on viral dynamics

All persons demonstrated significant decreases in HIV viral load at all measured time intervals. From week 0 to 12, participants demonstrated a median decrease of 2.7 log_10_ copies/mL (interquartile range [IQR] −3.59, −1.80; *P* ≤ 0.001), −3.55 log_10_ copies/mL (IQR −4.28, −1.80; *P* ≤ 0.001) between weeks 0 and 24, and −3.51 log_10_ copies/mL (IQR −4.44, −2.27) from week 0 to 48 (Supplemental Table 1). No differences in HIV viral dynamics were observed between the 2 groups. Similarly in the subset of study participants that HIV DNA was measured in, significant differences were noted overall but there were no differences between groups (Supplemental Table 2).

### 3.3 The impact of MVC intensification on circulating memory CD4^+^ T cells in acute and early HIV infection

All persons demonstrated statistically significant increases in CD4 T cell and CD4 percent at each measured time interval (Supplemental Table 1). The median CD4 T-cell gain from week 0 to 12 was 225 cells/mL (IQR 159, 339; *P* = 0.009); weeks 0 to 24 demonstrated a gain of 241 cells/mL (IQR 141, 328; *P* = 0.006), and weeks 0 to 48 revealed a difference of 326 (IQR 150, 356; *P* = 0.01). There were no differences in CD4 T-cell gains between SOC and MVC intensification.

**Figure 2:**
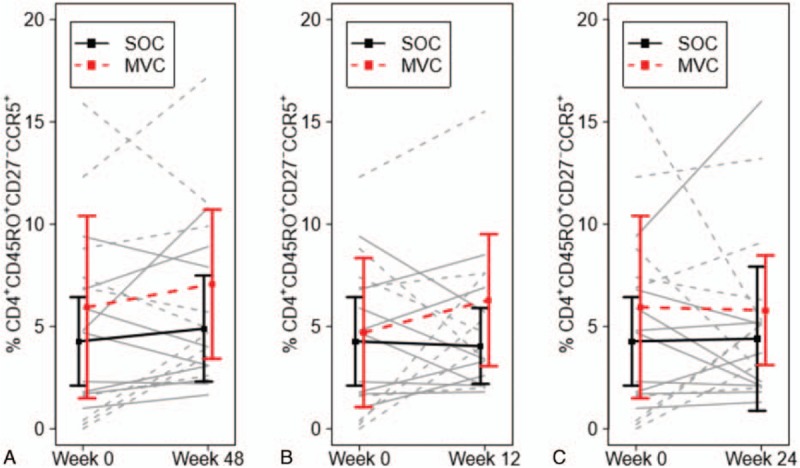
Changes in proportions of CCR5^+^ memory CD4 T cells in the blood were compared between persons starting SOC, ART (solid lines), and MVC intensification (dashed lines) in early HIV infection: (A) weeks 0 to 48, (B) weeks 0 to 12, and (C) weeks 0 to 24. Mean and 95% CI are represented for SOC (black) and MVC (red) arms. ART = antiretroviral therapy, CI = confidence interval, MVC = maraviroc, SOC = standard of care.

However, the primary outcome of the study was to evaluate whether the addition of MVC to SOC ART altered the proportion and dynamics of circulating plasma memory CD4^+^ T cells (CD4^+^CCR5^+^CD45RO^+^CD27^−^). In general, memory CD4^+^ T cells from baseline to week 48 increased, but there was no statistical difference between those who received MVC and those who did not (mean 1.12 vs 0.63, t = 0.36, DF = 16, *P* = 0.727) (Fig. 2). Incorporating baseline CD4^+^ T cells into the analysis of covariance model did not impact results. HIV viral load and HIV DNA were not incorporated into the model, but there were no differences in the rate of change of either of these values between the 2 arms at weeks 24 and 48 (Supplemental Tables 1 and 2).

### 3.4 The impact of MVC intensification on CD4^+^ and CD8^+^ T-cell dynamics in acute and early HIV infection

To determine whether CCR5 blockade with MVC impacted other CD4^+^ and CD8^+^ T cells, we performed exploratory analyses of multiple lymphocyte subsets including different maturation states (naïve, central memory, effector memory, and effector), coreceptor expression that impacts T-cell trafficking, activation state, cellular proliferation, and regulatory T cells in the peripheral blood (Table 2). We observed several trends in univariate analyses in both CD4^+^ and CD8^+^ T-cell subsets between the 2 groups over time. From baseline to week 12, the MVC group had greater increases in CD4^+^ naïve cells (CD45RO^−^CD27^+^CCR7^+^) (median 5.9% vs −1.5%, *P* = 0.112) and activated and proliferating CD4^+^ memory cells (CD45RO^+^CD27^+^HLA-DR^+^CD38^+^ and CD45RO^+^CD27^+^Ki67^+^) (median 0.7% vs −0.9% and 0.1% vs −2.5%, both *P* = 0.194).

**Table 2:**
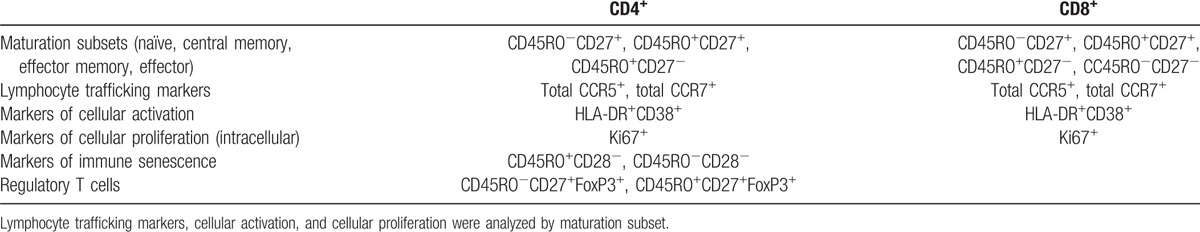
List of T-cell subsets evaluated in exploratory analyses.

From baseline to week 24, the MVC group had greater decreases in median CD4^+^ FoxP3^+^ naïve T cells (CD45RO^−^CD27^+^FoxP3^+^) (median −0.3% vs 0%, *P* = 0.143) and senescent memory CD4^+^ T cells (CD45RO^+^CD28^−^) (median −3.1% vs −0.1%, *P* = 0.064). Also at week 24, the MVC group still had higher naïve CD4^+^ T cells (median 4.8% vs −1.8%, *P* = 0.064). By week 48, all trends disappeared.

For CD8^+^ T cells, the MVC group had a slower decline in activated CD8^+^ T cells (CD45RO^+^CD27^+^HLA-DR^+^CD38^+^) from baseline to week 12 (median −0.7% vs −18.9%, *P* = 0.136). At week 48, the MVC group demonstrated slower increases in CD8^+^ naïve cells (CD45RO^−^CD27^+^CCR7^+^) (median 0.4% vs 7.8%, *P* = 0.158), and slower decreases in memory cells (CD45RO^+^CD27^+^) (median −8% vs −30.7%, *P* = 0.133) and activated memory cells (CD45RO^+^CD27^+^HLA-DR^+^CD38^+^ and CD45RO^+^CD27^+^Ki67^+^) (median −15.8% vs −29.9%, *P* = 0.133 and −10.9% vs −22.6%, *P* = 0.093).

## 4 Discussion

A previous study of MVC intensification in persons with recent infection (median time from infection to ART of 4 months) also demonstrated a faster rise in CD4^+^ T-cell counts and a slower decrease in CD8^+^ and CD4^+^ T-cell activation (CD38^+^HLA-DR^+^) in the MVC arm.^[15]^ Another study of MVC intensification during chronic infection demonstrated increases in activated CD8^+^ T cells and a less rapid decline in activated CD4^+^ T cells in the peripheral blood among those receiving MVC. In this study, rectal tissues were also sampled and showed increases in activated CD4^+^ and CD8^+^ T cells, suggesting that the peripheral findings were not due to altered trafficking into effector sites. The authors hypothesized that observed increases in activated T cells may be due to increases in plasma CCR5 ligands that result from MVC blockage of the CCR5 receptor.^[16]^

In summary, our study evaluated MVC intensification in persons initiating ART within 3 months of EDI. While all participants experienced increases in circulating memory CD4^+^ T cells, there were no significant differences between those who received MVC and those who did not. At most, MVC intensification of SOC ART led to weak trends of higher increases in naïve CD4^+^ T cells and slower declines in activated CD4^+^ and CD8^+^ T cells and decreases in senescent memory CD4^+^ T cells. This could have been related to differences in viral decay kinetics, but we did not observe differences in HIV viral load changes between arms. Limitations of our study include a limited number of study participants, lack of evaluations of gastrointestinal mucosa, the performance of multiple tests without a multiple comparison correction, and baseline group difference even with a randomized trial design. Additionally, performance of plasma CCR5 ligands in this population was beyond the scope of the study. Despite a few trends, there is no evidence to suggest that intensification of standard ART with MVC during acute HIV alters T-cell dynamics significantly in the peripheral blood. This suggests that diagnosis of early HIV and timing of ART may have greater clinical significance than the ART regimen used.

## Acknowledgments

Many thanks to our study participants and additional study-associated staff including Tari Gilbert, Celsa Spina, Neal Sekiya, Dina Sirypango, and DeeDee Pacheco without whom none of this work could have been completed.

## Supplementary Material

Supplemental Digital Content
