# Supplementary material for: A randomized controlled clinical trial on the impact of CCR5 blockade with maraviroc in early infection on T-cell dynamics
Source: Medicine (Baltimore). 2016 Nov 4;95(44):e5315. doi: 10.1097/MD.0000000000005315 (PMC5591160; doi:10.1097/MD.0000000000005315)
Supplement: Supplemental Digital Content [file medi-95-e5315-s001.docx]

Supplementary Figure 1. Flow Gating Example

CD4 and CD8 T cell subsets (CD27 CD45RO)


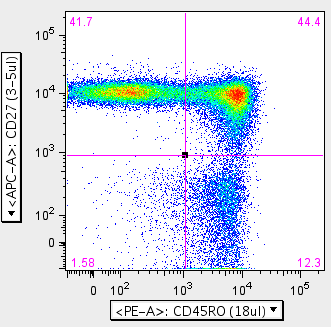


CCR5 expression by CD4 and CD8 maturation subsets

CD45RO^-^CD27^+^

CD45RO^+^CD27^+^

CD45RO^+^CD27^-^

CD4 and CD8 T cell differentiation

T cell by CD3

Lymphocytes by size


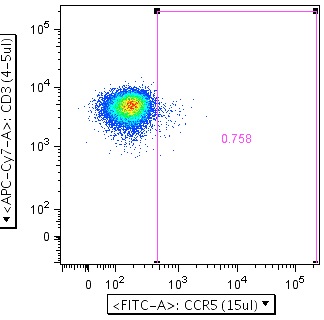


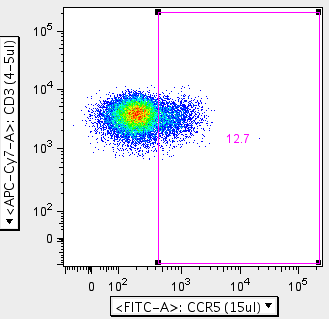


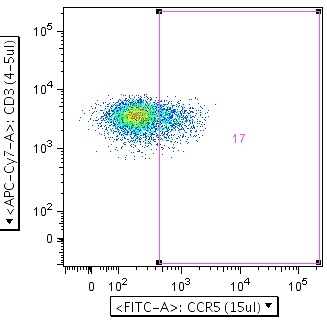


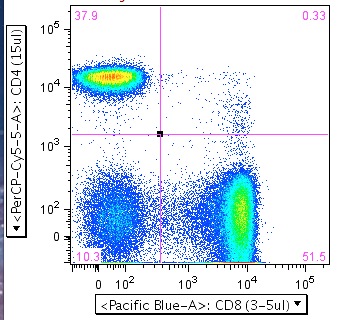


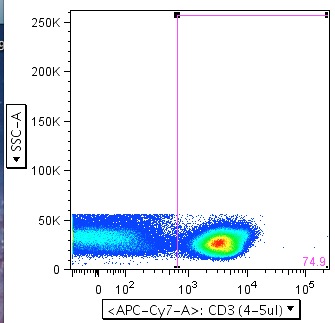


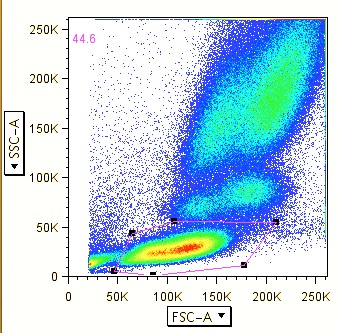


| *Supplemental Table 1. Change in Total CD4 T cells, CD4% and HIV viral load in all Participants and by Groups* | | | | | |
| --- | --- | --- | --- | --- | --- |
| *Measure* | *CD4 (Cells/μL)* | | | | |
|  | *Overall Change* | | *Group Comparison of Changes* | | |
|  | *All* | *P-value ^a^* | *SOC* | *MVC* | *P-value ^a^* |
| *Week 12 – Week 0* | 225 [159, 339], N=11 | **0.009**** | 225 [190, 326], N=5 | 283 [152, 364], N=6 | 0.58 |
| *Week 24 – Week 0* | 241 [141,328], N=10 | **0.006**** | 182 [132, 226], N=4 | 320 [178, 391], N=6 | 0.35 |
| *Week 48 – Week 0* | 326 [150, 356], N=11 | **0.01**** | 172 [166, 326], N=5 | 356 [136, 399], N=6 | 0.43 |
| *Week 24 – Week 12* | 45 [-38, 83], N=18 | 0.24 | 15 [-70, 46], N=8 | 53 [-23, 171], N=10 | 0.14 |
| *Week 48 – Week 12* | 11 [-39, 102], N=19 | 0.31 | 1 [-24, 45], N=9 | 68 [-52, 115], N=10 | 0.72 |
| *Week 48 – Week 24* | -48 [-100, 110], N=18 | 0.90 | 39 [-46, 193], N=8 | -65 [-134, 68], N=10 | 0.10 |
| *Measure* | *CD4 %* | | | | |
|  | *Overall Change* | | *Group Comparison of Changes* | | |
|  | *All* | *P-value ^a^* | *SOC* | *MVC* | *P-value ^a^* |
| *Week 12 – Week 0* | 10.0 [3.0, 15.5], N=11 | **0.023*** | 15.0 [10.0, 16.0], N=5 | 3.0 [2.3, 8.3], N=6 | 0.08 |
| *Week 24 – Week 0* | 7.0 [2.5, 11.5], N=10 | **0.049*** | 11.0 [8.8, 13.0], N=4 | 3.0 [1.3, 7.8], N=6 | 0.17 |
| *Week 48 – Week 0* | 9.0 [5.5, 14.0], N=11 | **0.029*** | 9.0 [9.0, 19.0], N=5 | 5.5 [4.3, 8.3], N=6 | 0.17 |
| *Week 24 – Week 12* | 0.5 [-1.0, 3.8], N=18 | 0.19 | 0.5 [-0.3, 4.3], N=8 | 0.5 [-1.0, 2.5], N=10 | 0.65 |
| *Week 48 – Week 12* | 1.0 [-1.5, 5.0], N=19 | 0.30 | 0.0 [-1.0, 4.0], N=9 | 1.5 [-1.8, 5.5], N=10 | 0.81 |
| *Week 48 – Week 24* | -1.5 [-3.0, 1.8], N=18 | 0.35 | -2.5 [-4.3, -0.5], N=8 | 0.0 [-3.0, 3.5], N=10 | 0.17 |
| *Measure* | *HIV RNA Viral Load (log_10_* copies/mL*) ^b^* | | | | |
|  | *Overall Change* | | *Group Comparison of Changes* | | |
|  | *All* | *P-value ^a^* | *SOC* | *MVC* | *P-value ^a^* |
| *Week 12 – Week 0* | -2.70 [-3.59, -1.80], N=19 | **<0.001**** | -3.09 [-3.24, -2.10], N=9 | -2.49 [-3.73, -1.68], N=10 | 0.90 |
| *Week 24 – Week 0* | -3.55 [-4.28, -2.27], N=19 | **<0.001**** | -3.15 [-4.14, -2.45], N=9 | -3.59 [-4.33, -2.23], N=10 | 0.90 |
| *Week 48 – Week 0* | -3.51 [-4.44, -2.27], N=19 | **<0.001**** | -3.15 [-4.14, -2.45], N=9 | -3.53 [-4.59, -2.23], N=10 | 0.84 |
| *Week 24 – Week 12* | -0.48 [-1.00, 0.00], N=19 | **0.003**** | -0.21 [-0.96, 0.00], N=9 | -0.52 [-1.03, 0.00], N=10 | 0.90 |
| *Week 48 – Week 12* | -0.48 [-1.04, 0.00], N=19 | **0.003**** | -0.21 [-0.96, 0.00], N=9 | -0.52 [-1.06, 0.00], N=10 | 0.97 |
| *Week 48 – Week 24* | 0.00 [0.00, 0.00], N=19 | 0.36 | 0.00 [0.00, 0.00], N=9 | 0.00 [0.00, 0.00], N=10 | >0.99 |

Note: Change in values is given as median difference [interquartile range] in each measure. Positive changes indicate increase in values over time; negative changes indicate decrease in values over time. N is sample size for available data.

^a^ Wilcoxon nonparametric test was used.

^b^ Changes were calculated on log_10_-transformed values; limit of detection was set at 50.

*p<0.05; **p<0.01. Significant p-values are in bold.

SOC – standard of care, MVC – maraviroc intensification

| *Supplemental Table 2: Differences in HIV DNA over time* | | | | | |
| --- | --- | --- | --- | --- | --- |
| *Measure* | *Week 12 – Week 0* | | | | |
|  | *Overall Change* | | *Group Comparison of Changes* | | |
|  | *All* | *P-value ^b^* | *SOC* | *MVC* | *P-value ^b^* |
| *HIV DNA (log_10_ copies/10^5^ CD4^+^T cells) ^a^* | -0.93 [-1.25, -0.76], N=14 | **0.003**** | -0.93 [-1.22, -0.87], N=6 | -0.88 [-1.15, -0.56], N=8 | 0.56 |
| *Measure* | *Week 24 – Week 0* | | | | |
|  | *Overall Change* | | *Group Comparison of Changes* | | |
|  | *All* | *P-value* | *SOC* | *MVC* | *P-value* |
| *HIV DNA (log_10_ copies/10^5^ CD4^+^T cells) ^a^* | -1.14 [-1.93, -0.54], N=14 | 0.09 | -1.41 [-1.98, -1.32], N=6 | -0.55 [-1.09, 0.44], N=8 | 0.06 |
| *Measure* | *Week 48 – Week 0* | | | | |
|  | *Overall Change* | | *Group Comparison of Changes* | | |
|  | *All* | *P-value* | *SOC* | *MVC* | *P-value* |
| *HIV DNA (log_10_ copies/10^5^ CD4^+^T cells) ^a^* | -1.17 [-1.49, -0.69], N=12 | **0.007**** | -1.30 [-1.52, -0.81], N=5 | -1.04 [-1.43, -0.58], N=7 | 0.42 |

Note: Change in values is given as median difference [interquartile range] in each measure from week 0 to each of weeks 12, 24, and 48. Positive changes indicate increase in values over time; negative changes indicate decrease in values over time. N is sample size for available data as many samples had undetectable HIV viral loads and undetectable HIV DNA.

^a^ Changes were calculated on log_10_-transformed values.

^b^ Wilcoxon nonparametric test was used.

*p<0.05; **p<0.01. Significant p-values are in bold.

SOC – standard of care, MVC – maraviroc intensification
